# Near Infrared-Triggered Liposome Cages for Rapid, Localized Small Molecule Delivery

**DOI:** 10.1038/s41598-020-58764-3

**Published:** 2020-02-03

**Authors:** Jeong Eun Shin, Maria O. Ogunyankin, Joseph A. Zasadzinski

**Affiliations:** 1 Department of Chemical Engineering and Materials Science, University of Minnesota, Minneapolis, Minnesota 55455 USA; 2 Present Address: Bristol, Myers, Squibb, 1 Squibb Drive, New Brunswick, NJ 08902 USA

**Keywords:** Biological techniques, Nanoscience and technology

## Abstract

Photolabile chelating cages or protecting groups need complex chemical syntheses and require UV, visible, or two-photon NIR light to trigger release. Different cages have different solubilities, reaction rates,  and energies required for triggering. Here we show that liposomes containing calcium, adenosine triphosphate, or carboxyfluorescein are tethered to plasmon-resonant hollow gold nanoshells (HGN) tuned to absorb light from 650–950 nm. Picosecond pulses of near infrared (NIR) light provided by a two-photon microscope, or by a stand-alone laser during flow through microfluidic channels, trigger contents release with spatial and temporal control. NIR light adsorption heats the HGN, inducing vapor nanobubbles that rupture the liposome, releasing cargo within milliseconds. Any water-soluble molecule can be released at essentially the same rate from the liposome-HGN. By using liposomes of different composition, or HGN of different sizes or shapes with different nanobubble threshold fluences, or irradiating on or off resonance, two different cargoes can be released simultaneously, one before the other, or in a desired ratio. Calcium release from liposome-HGN can be spatially patterned to crosslink alginate gels and trap living cells. Liposome-HGN provide stable, biocompatible isolation of the bioactive compound from its surroundings with minimal interactions with the local environment.

## Introduction

Probing physiological processes requires delivering bioactive molecules such as calcium, adenosine triphosphate (ATP), and other small, water-soluble molecules with sub-micrometer spatial and millisecond temporal resolution. The challenge is to locally perturb a specific pathway or process with a pulse of a biologically active molecule at a particular time and place^1–3^. The current strategy, called “caging” by J. F. Hoffman, who developed the first “caged ATP” in 1978^4^, involves the modification of a biologically active molecule with a photolabile protecting group that renders the “caged compound” inactive. The caged compound is added to the system being investigated and the bioactive molecule is cleaved from the protecting group with a pulse of light, rendering the molecule biologically active. A variety of neurotransmitters, nucleotides, peptides and enzymes have been caged by photolabile chromophores^1^. One limitation is that chromophores in the protecting group must contain multiple unsaturated carbon-carbon or carbon-nitrogen bonds and are inherently hydrophobic; hence, caging can decrease solubility. As a practical matter, each caged compound requires a separate synthesis with an appropriate photolabile caging group, which can be difficult. However, a number of caged compounds are available commercially^1^.

Inorganic ions such as calcium or magnesium cannot be directly caged, but can be trapped in photolabile derivatives of high-affinity chelators (BAPTA, EDTA and EGTA). Photolysis decreases ion affinity, releasing some fraction of the chelated ion^5,6^. As any chelator has a finite dissociation constant, there is always free calcium ion and free unloaded cage present. The higher the chelator affinity, the more ion that can be chelated before the free ion reaches an unacceptable level. To produce a net release of ion, all of the unloaded chelators must be photolyzed, otherwise the chelators simply re-complex the photo-released ions. Other divalent ions near the cage can be taken up by the chelating agent, which then can release the bioactive ion prematurely^1^.

To “uncage” the bioactive species requires breaking a covalent bond, which requires a significant energy input, typically associated with high energy ultraviolet (UV) light. Caged compounds with nitroaromatic chromophores are activated by light in the 340–410 nm range^1,4–6^. Advances in cage compound chemistry have moved the absorption maxima to 440–460 nm^3,7,8^. The requirement for near-UV excitation results in potential photodamage to other sensitive molecules, and the high scatter and light adsorption at UV wavelengths eliminates spatial localization beyond 1–2 cell diameters in biological media.

## Near Infrared Light Triggering – Possibilities and Problems

To address these limitations, Webb and Tsien^9^ proposed two-photon excitation using near infrared light (650–1000 nm) to uncage compounds. Near-IR light has greatly reduced scattering and adsorption in biological materials relative to UV or visible light^10^, increasing the penetration depth to hundreds of microns or more. The commercialization of two-photon microscopes^11^ makes delivering NIR light with cell level resolution accessible to most labs^12,13^. However, covalent bonds still require the equivalent energy of a UV photon to break. To achieve this energy, two-photon excitation requires the simultaneous absorption of two NIR photons of approximately half the energy of the equivalent UV photon. The rate of excitation per molecule is *R* = 1/2*σ*_2_〈*I*^2^〉, in which *σ*_2_ is the two-photon absorption cross-section in units of cm^4^-s-photon^−1^ (10^−50^ cm^4^-s-photon^−1^ equals 1 GM or Goppert-Mayer) and 〈*I*^2^〉 is the time average of the square of the incident local intensity in units of photons-cm^−2^-s^−1^. The two-photon microscope uses crossed light beams to obtain the necessary photon intensity at the focal spot to achieve spatial control of the release^1,2,11^. This relative rate of photolysis also depends on the volume of the focal spot as the residence time of a diffusing molecule within the two-photon volume is typically less than a millisecond; the chromophore can diffuse out of the high intensity focal spot before the reaction can take place.

However, photolysis via two-photon excitation is much less efficient than one photon excitation. The uncaging efficiency, *δ*_*u*_, is given by the product of the adsorption cross-section and the quantum yield of the uncaging reaction *ϕ*_*u*_; *δ*_*u*_ = *σ*_2_*ϕ*_*u*_. While there have been a plethora of new designs for two-photon cages^2,3,7,8,14–17^, few have the necessary high photolysis efficiency and good biological and solubility properties to replace UV triggered cages^2,14^.

## Universal Liposome-Hollow Gold Nanoshell “Cages”

An alternative to molecule-specific chemical caging is the rapid release of unaltered ATP or unchelated calcium (or other small molecules) trapped in unilamellar liposomes conjugated to hollow gold nanoshells (HGN). Release from the liposome is triggered by picosecond NIR light pulses that rapidly heat the HGN, leading to the formation of nanobubbles that rapidly expand and contract (similar to cavitation bubbles in ultrasound), mechanically lysing the liposomes. The liposome membrane provides a universal “cloak” to sequester the cargo of interest, protect against degradation and prevent premature release. Verma *et al*. have shown that liposome-encapsulated ATP remains 85% active compared to <0.5% free ATP after 25 minutes of exposure to ATP-ase^18^. We find that ATP can be held in liposomes for over a month without detectable release using a commercial ATP Determination Kit, which is based on the luciferin-luciferase bioluminescence assay. Ion permeation (especially divalent ions) through liposome membranes is extremely slow due to the change in solvation energy of the ion as it passes from the high dielectric constant of water to the low dielectric constant of the bilayer interior. In our hands, 5–50 mM calcium can be held in liposomes for over a month without detectable leakage by incubating the liposomes with the fluorescent dye Oregon Green BAPTA-1, which has a minimum detection level of 150 nM. No fluorescence was detected for over one month.

To create a universal delivery system, plasmon-resonant, hollow gold nanoshells (HGN) are covalently bound to the liposome exterior via DSPE-polyethyleneglycol (PEG)-thiol incorporated into the liposome membrane^19–21^. The plasmon resonance wavelength of the HGN can be tuned over the entire NIR wavelength range by modifying the ratio of the nanoshell wall thickness to the overall nanoshell dimensions (See Supplemental Information)^21,22^. The hollow shell structure moves the local surface plasmon resonance to NIR wavelengths^21^, so HGN absorb NIR light directly, and there is no need for high energy UV or two-photon absorption processes to break covalent bonds, which greatly increases the optical efficiency, while minimizing cell damage. Release is initiated by irradiating the liposome-HGN with picosecond or shorter NIR light pulses; the adsorbed light converts into heat causing the temperature of the HGN to increase hundreds of degrees over the first tens of nanoseconds following light adsorption^19–21,23^ before any significant temperature change can occur in the surrounding liquid^19–21,24–27^. In the following nanoseconds, however, the HGN dissipates its thermal energy by vaporizing a minute amount of water, nucleating transient nanobubbles around the HGN^19–21,23–29^.The nanobubbles rapidly expand and collapse, similar to cavitation bubbles formed by ultrasound^19^, without significantly changing the bulk liquid temperature. The mechanical forces caused by the growth and collapse of the nanobubbles ruptures the adjacent liposome membrane, and the cargo is jettisoned from the liposome with micron spatial resolution and millisecond time resolution^12,13,19,20,30–33^. Triggered release can be accomplished within individual cells following endocytosis of liposome-HGN with sub-cell level resolution^12,13,30–34^. The released amount and the release rate  are dependent only on the plasmonic properties of HGN, the mechanical properties of liposomes, and the wavelength and fluence of the NIR light, not on the chemical nature of the cargo. We show here that release can be determined by controlling (1) the plasmon resonance wavelength relative to the laser wavelength by tethering HGN with different sizes and/or shell thicknesses to the liposome; or (2) controlling the liposome composition to adjust the lysis tension and mechanical properties of the liposome itself. Different light fluences can be used to control the release rates of each liposome cargo, providing spatial and temporal control of biomolecule release. Any lab with access to two-photon microscopes which can supply the necessary pulsed NIR light of controlled wavelength could use this technique as the synthesis of HGN is simple and liposomes are common to many biological labs, especially in comparison to the organic synthesis necessary to make a UV or two-photon caged compound.

## Results and Discussion

**Figure 1:**
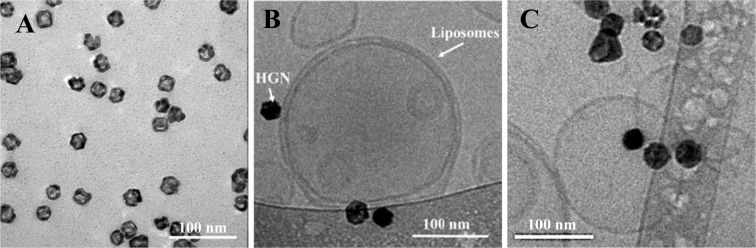
(**A**) Hollow gold nanoshells take on the cubic shape of the silver crystal template (SI Fig. S1) but with a hollow interior. The ratio of wall thickness to cube dimension determines the SPR wavelength^21,22^. (**B**) TEM image of carboxyfluorescein containing DPPC liposome with HGN attached via a DPPE-PEG-thiol tether. (**C**) DPPC liposomes with ATP showed the typical smooth spherical shape with HGN attached. This image were taken one month after the liposomes were prepared and shows no difference with (**B**) which was prepared one day after preparation.

Hollow gold nanoshells (HGN) synthesized by galvanic replacement of solid silver nanocrystal template (Supplemental Information) by gold (III) chloride hydrate (Fig. 1A)^21,22,35^ were tethered to dipalmitoylphosphatidylcholine (DPPC) or DPPC-cholesterol ( 55:40 mol:mol) liposomes containing 4–5 mol % distearoylphosphatidylethanolamine linked to a 2000 Da PEG terminated in a thiol (DSPE-PEG-2000-SH; Nanocs, New York, NY) via the thiol-gold bond (Fig. 1B,C) (See Supplemental Information for details). The local surface plasmon resonance (LSPR) red-shifts from 420 nm to 650–900 nm depending on the ratio of gold salt to silver during the galvanic replacement reaction (Supplemental Information). Liposomes containing biomolecules of interest were prepared by the standard thin film hydration/extrusion method. Conditions were chosen to insure 1–2 HGN per liposome on average (Details in Supplemental Information) (Fig. 1B,C). HGN/liposomes are stable against aggregation in saline or serum for weeks^36,37^.

**Figure 2:**
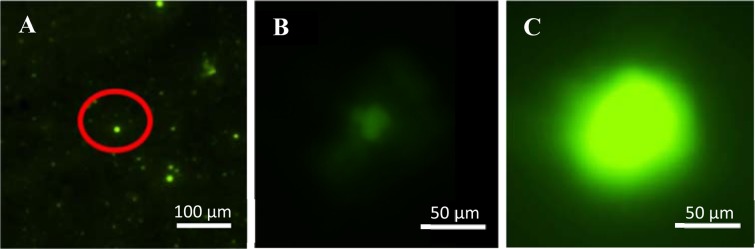
(**A**) Resolution-limited confocal fluorescence image (emission maxima for CF is 517 nm with excitation at 495 nm) of individual liposome-HGN containing 25 mM quenched CF attached to a polylysine-coated glass slide. (**B**) Higher magnification image of the isolated liposome-HGN in the red oval in (A). Prior to NIR irradiation in the two-photon microscope, the fluorescence is quenched at the high internal concentration of the intact liposome. The NIR laser in the two-photon microscope is simply a source of pulsed NIR light; no combination of the NIR photons to create a higher energy UV or visible photon is necessary for release. (**C**) After NIR irradiation, the CF dye is released and is diluted as it spreads, increasing the CF fluorescence intensity at 517 nm a few milliseconds after irradiation as shown by conventional confocal fluorescence microscopy.

Figure 2A shows confocal microscope images of carboxyfluorescein (CF) containing liposome-HGN adsorbed to a glass slide treated with polylysine taken in a combined two-photon/confocal microscope. The red circle outlines a single, resolution-limited image of a CF liposome, shown at higher magnification in Fig. 2B. Prior to irradiation, the CF fluorescence is predominantly quenched at the high concentrations present in the intact liposomes. However, when the pulsed 800 nm NIR light is focused on the individual liposome, the liposome-HGN was ruptured and CF was released to the surrounding solution. As the CF is diluted on being released from the liposome, Fig. 2C shows the localized increase in fluorescence intensity around the specific liposome within 1 msec following the irradiation^34^. None of the other liposomes in Fig. 2A were ruptured; as with conventional caged compounds, release from liposome-HGN can be spatially localized by focusing the beam of the two-photon microscope^12^.

### HGN size effects on release

**Figure 3:**
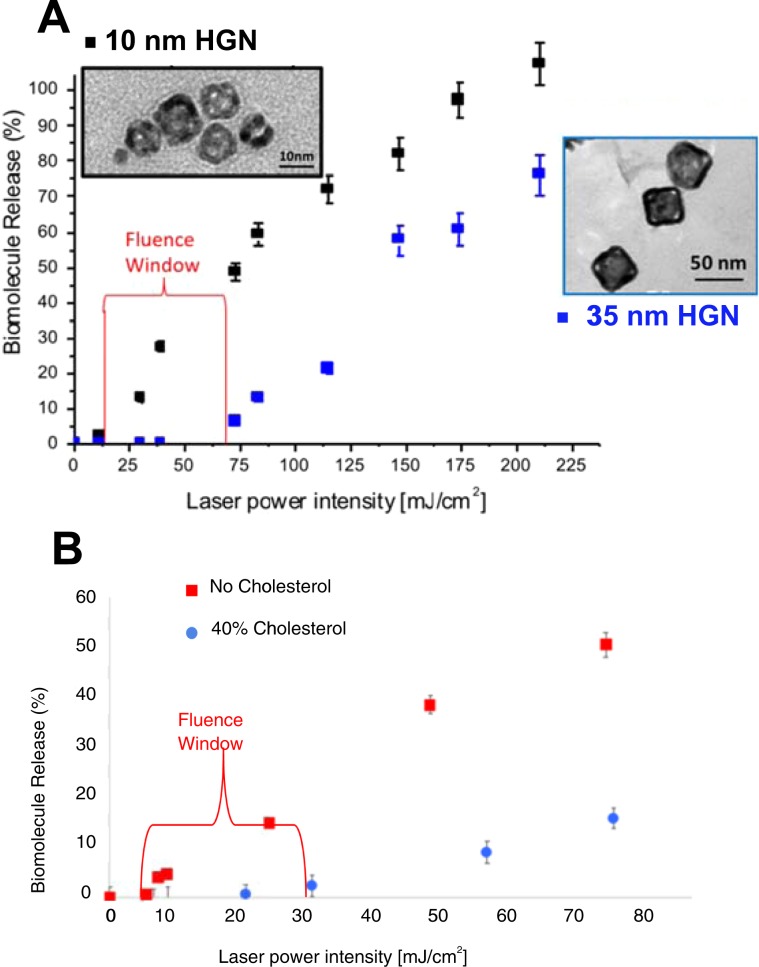
(**A**) 10 nm HGN with a LSPR of 800 nm rupture DPPC liposomes at ~10 mJ/cm^2^, while 35 nm HGN, also with a LSPR of 800 nm, require ~70 mJ/cm^2^ to induce rupture when irradiated with 800 nm NIR light. This creates a “fluence window” over which one liposome-HGN releases its contents while the second remains intact. Above 70 mJ/cm^2^, further increases in fluence showed proportional increases in calcium release for both sets of liposomes. (**B**) Calcium release from DPPC:DSPE-PEG 95:5 is initiated at ~10 mJ/cm^2^ compared to DPPC:Cholesterol:DSPE-PEG 55:40:5 which does not release until ~30 mJ/cm^2^. Adding cholesterol to DPPC liposomes increases the lysis tension, which in turn increases the fluence needed to release liposome contents.

Figure 3A shows that there is a significant difference in 800 nm laser fluence needed to release calcium from liposomes tethered to 10 nm or 35 nm diameter HGN; both HGN have been designed to have their LSPR maxima at 800 nm (see Supplemental Information). This creates a “fluence window” over which liposomes with 10 nm HGN are ruptured, while identical liposomes with 35 nm HGN are not^21^. The release of calcium from the liposomes was quantified by the increase in Oregon Green 488 BAPTA fluorescence intensity above the background relative to the fluorescence intensity after all liposomes were completely lysed^38^. For liposomes tethered to 10 nm HGN, calcium release begins at ~10 mJ/cm^2^ laser fluence, while >70 mJ/cm^2^ was required to initiate calcium release from liposomes tethered to 35 nm HGN. Over the “fluence window,” the 10 nm liposome-HGN released 50% of the contents, while the 35 nm liposome-HGN remained intact. Increasing the fluence above 75 mJ/cm^2^ increased the fractional calcium release with similar slopes for both liposome-HGN. Below the minimal fluence, no calcium release was observed for over 1 month. Liposomes without HGN did not show any observable change in the calcium dependent Oregon Green 488 BAPTA fluorescence intensity on irradiation at any fluence, nor did liposome/HGN prior to NIR irradiation.

#### Liposome composition effects on release

Mechanical forces due to the cavitation induced by nanobubble formation and collapse following NIR absorption is responsible for the transient membrane rupture leading to contents release^19,20^. Hence, bilayer rupture likely depends on the mechanical properties of liposome membrane. Needham and Nunn^39^ showed that adding cholesterol to phospholipid bilayers increased the lysis tension at which the bilayer membrane failed; a maximum in liposome toughness occurred at 40 mol% cholesterol. This suggests that DPPC-cholesterol liposomes should require larger cavitation forces to rupture, and a corresponding higher laser fluence.

To test this hypothesis, the same 10 nm HGN with a LSPR of 800 nm were tethered to the outer surface of liposomes of 95:5 DPPC:DSPE-PEG and to liposomes of the higher lysis tension concentration 55:40:5. DPPC:Cholesterol:DSPE-PEG^39^. Figure 3B shows that the liposomes without cholesterol required ~10 mJ/cm^2^ to initiate calcium release, while ~30 mJ/cm^2^ was required to initiate calcium release from liposomes containing 40% cholesterol, creating a 20 mJ/cm^2^ fluence window, confirming the hypothesis that higher lysis tension liposomes require higher laser fluences and larger cavitation forces.

#### Irradiating on and off resonance for sequential release

**Figure 4:**
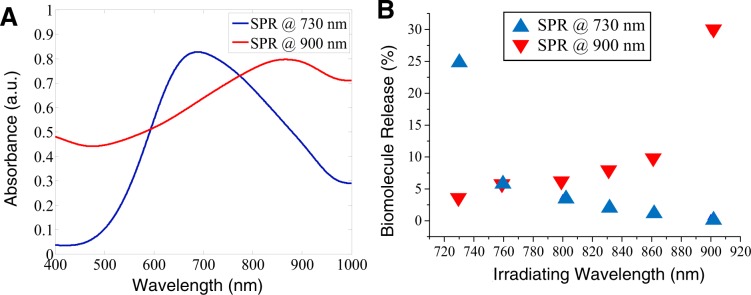
(**A**) Absorption spectra for 20 nm HGN with different wall thicknesses, leading to different SPR peaks at 730 nm and 900 nm. (**B**) Calcium release from otherwise identical liposomes tethered to the HGN whose spectra appears in (**A**). In both cases, irradiating the liposome-HGN at a constant fluence of 24 mJ/cm^2^ produces the maximum contents release when the irradiation wavelength is the same as the SPR. Content release drops off sharply as the irradiating wavelength moves off resonance for both liposome-HGN.

A convenient way to induce release from one set of liposomes without allowing release from a second set of liposomes is to use HGN with different LSPR. Previous work showed that HGN irradiated at their LSPR require the minimum fluence for nanobubble formation, and this threshold fluence sharply increases as the difference between the irradiating wavelength and the LSPR maximum increases^21,40^. Figure 4A shows the absorption spectra of two sets of 20 nm HGN of different wall thickness with SPR of 730 and 900 nm. The two sets of 20 nm HGN with different SPR were attached to two sets of otherwise identical CaCl_2_-containing 95:5 DPPC:DSPE-PEG liposomes and irradiated with a constant laser fluence of 24 mJ/cm^2^, but at different wavelengths. Figure 4B shows that when irradiated at their respective LSPR peaks, both sets of liposomes showed maximum contents release of 25–30%. Contents release for both liposome-HGN dropped by a factor of ~5 when the irradiation wavelength moved off the SPR as little as 30–40 nm. When liposome-HGN with an SPR peak of 730 nm were irradiated at 900 nm, no liposome contents were released; however, liposome-HGN with their SPR peak at 900 nm still released ~4% of contents. This is likely due to the broader spectra of these HGN that still showed appreciable adsorption at 700 nm (Fig. 4A), but could be minimized by decreasing the laser fluence.

#### ATP release from liposome-HGN

**Figure 5:**
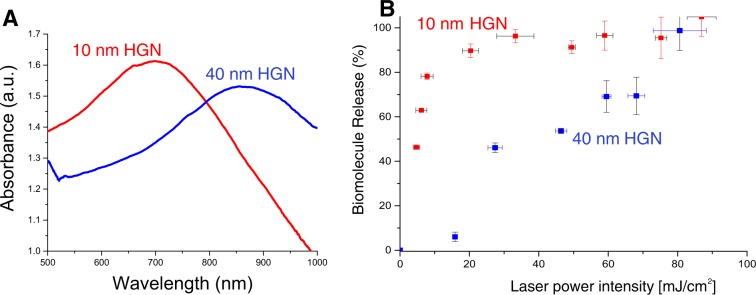
(**A**) Absorption spectra for 10 nm HGN and 40 nm with different SPR peaks at 730 nm and 880 nm, respectively. (**B**) ATP release from otherwise identical liposomes tethered to the HGN whose spectra appears in (**A**) irradiated with 730 nm light. ATP is almost completely released by a fluence of 20 mJ/cm^2^ from the on-resonance liposome-10 nm HGN, before any contents release occurs from the off-resonance liposome-40 nm HGN.

As release from liposome/HGN is induced by nanobubble -cavitation rupture of the liposome membrane, the release should be independent of the liposome cargo. Figure 5A shows the absorption spectra of 10 nm HGN with a SPR peak at ~730 nm and 40 nm HGN with a SPR peak at ~880 nm. Figure 5B shows that when these HGN are tethered to otherwise identical liposomes containing 10 mM ATP, irradiation with 730 nm light initiated release from the 730 nm SPR liposomes at about 8 mJ/cm2, while 20 mJ/cm2 was necessary to release ATP from the 880 nm SPR liposomes. Combining the fluence differences due to on and off resonance irradiation with the differences due to the HGN size led to almost complete release from the 10 nm, on resonance liposome-HGN before any release occurred from the 40 nm, off resonance 40 nm -liposome HGN. Unlike conventional caged compounds, the chemical features of the caged compounds do not have an appreciable effect on the release rates from the liposome-HGN; ATP and calcium are released from liposome-HGN by the same nanobubble rupture process; compare Figs. 4 to 5.

#### Mixed liposomes – controlling  ATP to calcium ratio

**Figure 6:**
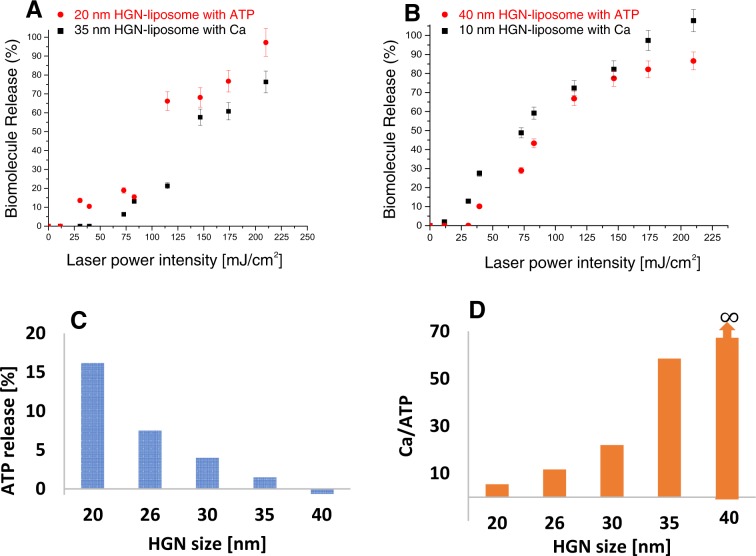
(**A**) Release from mixed liposome-HGN containing ATP (red) or calcium chloride (black) tethered to 20 or 35 nm HGN respectively, both with 800 nm SPR and irradiated by 800 nm light. The ATP liposomes begin to release their contents at >15 mJ/cm^2^, while the Ca liposomes do not start to release until >50 mJ/cm^2^. Increasing the fluence releases more of the cargo and the ratio of ATP to calcium can be adjusted by increasing the fluence. (**B**) By placing the calcium chloride in the liposomes with 10 nm HGN and the ATP in the liposomes with 40 nm HGN, the release sequence is reversed. Calcium is released at ~13 mJ/cm^2^, while ATP does not start to release until ~40 mJ/cm^2^. Increasing the fluence also releases more of the cargo, but more calcium is released at all fluences. (**C**) Calcium-containing liposomes tethered to 10 nm HGN were mixed with equal volumes of ATP containing liposomes tethered to 20 nm, 26 nm, 30 nm, 35 nm, and 40 nm HGN with SPR of 800 nm. The mixtures were irradiated with 800 nm NIR pulses at 42 mJ/cm^2^. The ATP release decreased with increasing HGN size, dropping to 0 for 40 nm HGN. The calcium release was constant at 90%. (**D**) Ratio of calcium to ATP released increased with increasing HGN size from about 5:1 for the 20 nm HGN to infinity for the 40 nm HGN  for  a fluence of 42 mJ/cm^2^.

With the fluence windows made possible by controlling HGN size, SPR vs irradiation wavelength, and liposome composition, it is possible to control the sequence or time release of two liposome-HGN with different cargoes. Calcium release was determined by monitoring Oregon Green 488 BAPTA fluorescence intensity and ATP release was measured by monitoring luciferin-luciferase based bioluminescence intensity to avoid crosstalk between the calcium indicator and the ATP indicator. Figure 6A shows cargo release from a sample of mixed liposomes, one set with tethered 20 nm HGN containing ATP, and a second set with tethered 35 nm HGN containing calcium chloride. Both HGN were designed to have a SPR of ~800 nm and were irradiated with 800 nm light pulses. The 20 nm HGN-liposomes began to release their ATP cargo at a laser fluence >15 mJ/cm^2^, compared to ~50 mJ/cm^2^ for release of calcium from the 35 nm HGN/liposomes. Increases in fluence above 50 mJ/cm^2^ increased the release of both calcium and ATP similarly, so it was possible to vary the ATP/calcium ratio over a wide range. Figure 6B shows that the liposome cargo release profiles could be easily reversed to release calcium before ATP by sequestering calcium chloride in 10 nm HGN-liposomes and ATP in 40 nm HGN-liposomes. Now, calcium was released at ~13 mJ/cm^2^ and ATP was released at ~40 mJ/cm^2^, and further fluence increases could provide almost any desired ratio of calcium/ATP. Figure 6 shows that release is not dependent on the liposome cargo, but rather on the fluence necessary to rupture the liposomes via nanobubble formation. The order of contents release can easily be swapped, showing the flexibility of our HGN/liposome delivery platform.

Figure 6C shows the flexibility of the HGN-liposome release. Calcium-containing liposomes were tethered to 10 nm HGN, while ATP containing liposomes were tethered to 20 nm HGN, 26 nm HGN, 30 nm HGN, 35 nm HGN, and 40 nm HGN. The SPR peaks of all the HGN were at 800 nm and 800 nm NIR pulses at 42 mJ/cm^2^ were used. Equal volumes of calcium-containing HGN/liposomes and ATP containing HGN/liposomes were mixed together. The calcium to ATP release ratio was defined as [Ca release %]/[ATP release %]. The size of HGN tethered to calcium-containing liposomes was fixed at 10 nm, leading to ~90% calcium release in all mixtures. However, as shown in Fig. 6C, ATP release decreased significantly with increasing HGN size, for 40 nm HGN, the ATP release dropped to 0%. This provided an increase in the calcium to ATP release ratio of 5, 12, 22, 58, and ∞ for 20 nm HGN, 26 nm HGN, 30 nm HGN, 35 nm HGN, and 40 nm HGN, respectively (Fig. 6D).

**Figure 7:**
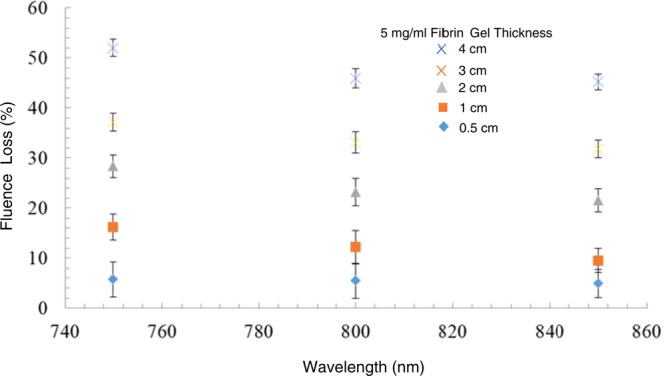
The decrease in fluence was measured for 5 mg/ml fibrin gels in glass vials of various thickness at 750, 800 and 850 nm. The maximum loss was about 50% for the 4 cm gel thickness. Fluence loss decreases slightly with increasing wavelength.

To demonstrate the penetrating power of NIR light, we measured the fluence loss through fibrin gels up to 5 cm thick. The fibrin gels were formed at 5 mg/ml fibrin concentration in glass vials; they were opaque to visible light. Figure 7 shows that the laser fluence was reduced by ~20% and ~50% after passing through 1 and 4 cm of 5 mg/ml fibrin gels. The loss decreased slightly at higher wavelengths.

**Figure 8:**
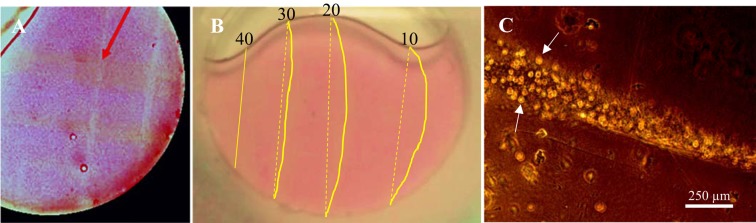
(**A**) 8 mm thick 8% w/v alginate gel irradiated in a square grid pattern to release calcium from liposome-HGN. The irradiation causes the gel to turn colors from pink to gold/white as the calcium dye Arsenazo III which turns red on complexation with calcium diffuses away. (**B**) 8 mm thick 8% w/v alginate gel irradiated along vertical lines (highlighted by dotted yellow lines) at 40, 30, 20 and 10 mJ/cm^2^ fluence of 800 nm pulsed NIR light to release calcium from liposome-HGN. The gel was placed on a 30 incline and the gel flowed (as indicated by the motion of the gold/white lines as in A) different amounts depending on the applied fluence (highlighted by solid gold lines). The greater the fluence, the more calcium was released locally, and the greater the stiffness of the gel. (**C**) PC cells were mixed homogeneously in a softly gelled 2% w/v alginate gel along with calcium containing liposome-HGN. 80 mJ/cm^2^ of 800 nm NIR light of beam diameter 300 µm was irradiated along lines in the gel to locally release calcium to stiffen the gel. The gel was washed with PBS and growth medium, and incubated for 48 hours in growth media. The growing and dividing PC cells (bright circular structures at arrows) were found primarily along the irradiated, stiffened lines, which appear bright in the image as the Arsenazo III dye originally in the liposome has diffused away.

Alginate is a natural polysaccharide extracted from brown seaweed that consists of mannuronic and guluronic monomers. The guluronic region can crosslink with divalent cations such as Ca^2+^; the stiffness of gel depends on the calcium concentration. We used 1000 nm liposome-HGN containing 500 mM calcium to stiffen 8% w/v alginate solutions partially gelled with 50 mM calcium chloride solution by locally increasing the calcium concentration by patterned irradiation of the alginate with 800 nm NIR light pulses (see Supplemental Information for details). To visualize the distribution of the calcium containing liposome-HGN, the calcium specific dye, Arsenazo III was added to the liposome contents. Arsenazo III complexes with calcium and exhibits a characteristic red color; prior to irradiation, the gel containing liposome-HGN is uniformly colored red. The gels were approximately 8 mm thick. Figure 8A shows a partially gelled alginate solution irradiated with NIR pulses at 140 mJ/cm^2^ at 800 nm wavelength to trigger release of calcium from the liposomes to crosslink the alginate solution in a square grid pattern of lines. Along the lines, the gel turns color from red to gold/white indicating the release of the calcium and dye from the liposomes, the calcium then diffuses into and complexes with the alginate and the dye dissipates. A qualitative indication of the increasing gel stiffness is shown in Fig. 8B. A similar 8% w/v alginate solution was partially gelled with 50 mM calcium chloride solution and mixed with 1000 nm liposome-HGN containing 500 mM calcium. Following irradiation along vertical lines (highlighted by yellow dotted lines in Fig. 8B to aid the eye) at fluences of 40, 30, 20, and 10 mJ/cm^2^, the gel was placed on a 30° incline and followed for 5 minutes. The solid gold lines (to aid the eye) in Fig. 8B highlight the flow of the gel, which was inversely proportional to the laser fluence. The gel irradiated at 40 mJ/cm^2^ did not flow at all, while the gel irradiated with 10 mJ/cm^2^ flowed the most, indicating that different amounts of calcium release were released locally by different light fluences, resulting in gels of different resistance to flow.

The stiffness of the extracellular matrix (ECM) has a significant effect on cell behavior, including cell migration, proliferation, morphology and differentiation^41,42^. To mimic the natural dynamic microenvironment of the ECM, hydrogels with tunable stiffnesses are required^42^. To show that the liposome-HGN calcium delivery is compatible with cell culture, a 2% (w/v) alginate solution in PBS and cell growth medium (RPMI1640) was mixed with PC3 prostate cancer cells and  500 mM calcium-containing liposome-HGN. The HGN size was 40 nm and the LSPR was 800 nm. The gel was then softly gelled with 100 mM calcium chloride in PBS. As before, pulsed NIR light at 800 nm at a fluence of 80 mJ/cm^2^ were applied along a grid pattern to locally release calcium from the liposome-HGN. The gel was then washed out with PBS and cell growth medium to remove any cells that were not anchored by the gel. The gel was incubated in cell growth medium for 48 hours to let cells grow. Figure 8C shows that the PC3 cells remained and grew primarily in the irradiated parts of gel after 48 hours of incubation. Cell viability, as indicated by cell division and growth, was maintained during stiffness modulation.

## Conclusions

The combination of liposomes for sequestration and protection of small biologically active molecules, with near infra-red pulsed light triggered rupture by nanobubble formation around hollow gold nanoshells, can provide spatially and temporally localized delivery of many different small molecules in physiologically relevant environments. This generic delivery platform separates the delivery mechanism from the delivered cargo, in contrast to conventional caged compounds. Each bioactive molecule requires the chemical synthesis of its own “cage”; as yet, few caged compounds can combine the necessary solubility and two-photon cross section and optical efficiency to be effective under typical experimental conditions with NIR excitation. HGN are activated directly by picosecond pulses of NIR light that can be delivered by conventional two-photon microscopes or by stand-alone lasers during microfluidic flow in capillaries or to pattern hydrogels or other media. NIR light can penetrate 1–4 cm of fibrin gels with sufficient light fluence to activate the HGN; the UV light needed to activate conventional caged compounds cannot penetrate more than 10–20 microns. We can even pattern alginate hydrogel stiffness to regulate the location of viable cells. We have shown that chemically disparate calcium, ATP, and carboxyfluorescein (CF) can be released at different ratios from different liposome-HGN combinations by controlling the HGN size, the liposome membrane composition, and by irradiating on or off resonance for different HGN.

A major benefit of this technique is the universal mechanism of liposome contents release via nanobubble rupture following pulsed NIR light triggering. Our HGN tethered liposomes are able to encapsulate almost any water-soluble biologically active molecule in relatively high concentrations. The nanobubbles release these water-soluble biomolecules by mechanical liposome rupture, so release rates, timing, laser fluence, etc. are similar for all compounds of interest. The release dynamics are not affected by the nature of cargo, but by the plasmonic properties of HGN and mechanical properties of liposomes. We can create “fluence windows” over which one set of liposomes can release their contents while a second set cannot, by controlling the HGN size, with 10 nm HGN releasing at lower fluence than 20–40 nm HGN, for example. We can also create fluence windows by tuning the SPR wavelength of the HGN relative to the wavelength of the irradiation. The fluence required to induce liposome rupture increases quickly as the irradiation wavelength is moved off the SPR. Finally, increasing the cholesterol fraction of DPPC liposomes increases the lysis tension, which, in turn, increases the fluence required to rupture the liposomes. This allows us to create liposomes that can release at different laser fluences to control release rates and create fluence windows for each biomolecule in a mixture independently, by delivering different combinations of liposome composition or HGN size or wall thickness. In this way, we can release one compound at one place and time, then a second compound at the same place at a different time simply by modulating the laser energy. Appropriate choice of HGN size and LSPR wavelength, as well as liposome composition allowed us to alter the energy threshold for triggering contents release and the combination with appropriate irradiating wavelength and laser fluence enabled us to trigger multiple contents independently and sequentially with the same generic delivery platform. Release can also be accomplished in hydrogel matrices due to the superior penetration of NIR light and can be used in cell cultures to pattern the distribution of cells in hydrogels.

## Experimental

### Materials

Gold(III) chloride hydrate (HAuCl_4_, ≥99.999%), sodium citrate (HOC(COONa)(CH_2_COONa)_2_ · 2H_2_O), sodium borohydride (NaBH_4_, powder, ≥98.0%), and hydroxylamine hydrochloride (NH_2_OH∙HCl, 99%) were obtained from Sigma-Aldrich (St. Louis, Mo). Silver nitrate (AgNO_3_, crystalline, ≥99.7%) was purchased from Fisher Scientific. Methoxy-PEG-thiol of 750 Da and distearoylphosphatidylethanolamine linked to a 2000 Da PEG terminated in a thiol, (DSPE-PEG-2000-SH), were purchased from Nanocs (New York, NY). Dipalmitoylphosphatidylcholine (DPPC) and cholesterol were purchased from Avanti Polar Lipids (Alabaster, Al). All chemicals were used as received. Water with a resistivity of 18.2 MΩ·cm at 25 °C was purified with a Millipore Direct Q 3UV-R (Billerica, MA) system.

### HGN synthesis

Hollow gold nanoshells (HGN) were synthesized by galvanic replacement of silver nanocrystal templates by gold (III) chloride hydrate as described in Ogunyankin *et al*.^21,22,35^ and described in detail in the Supplementary Information. As the gold replaces silver, the localized surface plasmon resonance (LSPR) red-shifts from 420 nm to 650–900 nm depending on the ratio of gold salt to silver (See Fig. S2). The LSPR is determined primarily by the ratio of the wall thickness to the overall dimensions of the HGN (See Fig. S3). 750 Da thiol terminated polyethylene glycol is grafted onto the HGN surface at a 1:10 PEG:gold (mol:mol) ratio to prevent aggregation of the HGN in normal saline. HGN are stable in saline for more than one month prior to linking with liposomes.

### Liposome-HGN

Liposomes containing 25 mM carboxyfluorescein (CF) in TES buffer, 10–50 mM calcium in PBS buffer, or 10 mM ATP in PBS buffer were prepared by standard thin film hydration/extrusion^21^. Briefly, 95:5 DPPC:DSPE-PEG-SH or 55:40:5 DPPC:cholesterol:DSPE-PEG-SH were mixed at 25 mg/ml total lipid concentration in chloroform, and the solvent was evaporated. The dried lipids were hydrated 30 minutes at 60 °C with either 25 mM CF, 10–50 mM CaCl_2_ or 10 mM ATP in buffer and extruded using an Avanti Mini-Extruder through Watson 200 nm polycarbonate filters. HGN were mixed with the thiol-PEG liposomes overnight at room temperature at relative concentrations to provide 1–3 HGN per liposome (See SI for details). Untethered HGN and unencapsulated CF, CaCl_2_ or ATP were removed by size-exclusion chromatography using a PD MidiTrap G-25 column (GE Healthcare) equilibrated with buffer at pH 7.4. The mean size of the liposomes was 180 nm determined by Nanosight particle tracking and cryo-TEM imaging and was constant over the course of 1 month.

### Two-Photon and confocal microscopy

The images and NIR irradiation in Fig. 2 were performed using a Nikon A1RHD confocal/two-photon microscope. Liposome-HGN were triggered with a mode-locked Ti:sapphire femtosecond pulsed laser (100 fs pulse duration, 80 MHz repetition rate, Spectra Physics 15 W Mai Tai eHP tunable IR laser) tuned to 800 nm at a raster scan speed of 125,000 Hz up to 35 full-frame cycles regulated with a modulator controlled by the microscope software. Spatially controlled release experiments were performed by selecting a region of interest using the microscope software to scan only the selected area with the femtosecond pulsed laser. Following excitation with the NIR laser, carboxyfluorescein fluorescence was imaged with a 15 mW blue laser diode exciting at 473 nm raster scanning at a speed of 80,000 Hz in conventional confocal fluorescence mode.

### Laser irradiation: nanobubble generation and detection in flow

For the calcium and ATP release experiments, 28 picosecond pulses of near infrared light of wavelengths 650–1000 nm of controlled fluence at a 50 Hz repetition rate were generated by pumping a PG403 optical parametric generator with a PL 2231 pulsed laser at 355 nm (Ekspla, Vilnius, Lithuania). A continuous wave Helium-Neon probe laser (632.8 nm, 2 mW, polarized, Thorlabs, Inc.) focused at the same spot as the pulsed laser was used to detect nanobubble generation. The pump and probe beams were focused onto a 0.2 mm ID square, hollow glass capillary of 0.1 mm wall thickness (#8320 Vitro Tubes, VitroCom, Mountain Lakes, NJ). Silicone tubing (Ibidi GmbH., I.D. 0.5 mm, wall thickness 0.8 mm) connected the optically transparent glass capillary to a programmable syringe pump (Harvard Apparatus, Holliston, MA). The pump beam diameter was set to 300 µm and was tilted at 15° so the pump beam would miss the lens used to collect the light from the probe beam, which was aligned normal to the capillary. The suspension flow rate through the capillary was 18 µl/min, providing an average velocity of 3.8 mm/sec; matching the beam diameter and the laser pulse rate of 50 Hz gave an average of two pulses to any particular liposome. The pump beam flux was measured by registering the image of the pump beam on the capillary and measuring the beam diameter at the sample plane with a photodetector/imaging device (Luka, Andor Technology, Northern Ireland). The pulse energy was measured using a pulse energy meter (Ophir Optronics, Ltd., Israel). The measured temperature increase following irradiation was <0.5 °C. After irradiation, the HGN suspension was passed through silicone tubing and was collected in vials for analysis of liposome contents release. Operation of all hardware was controlled by a PC through custom software modules developed using the LabVIEW platform.

The continuous probe beam passed through the capillary onto a photodetector and the signal was collected by an oscilloscope (Wavesurfer MXs-8,Teledyne LeCroy)^43^. The probe beam is scattered by nanobubble formation, decreasing the probe beam intensity that reaches the photo-detector, providing a characteristic signal of nanobubble generation and growth. Nanobubbles are transient events, lasting a few hundred nanoseconds. Release from liposomes was directly correlated with nanobubble formation^21^.

For gel patterning experiments, the partly gelled alginate solution in a petri dish was irradiated with a pump beam diameter of 300 µm with 800 nm NIR light at various fluences by manual displacement of the petri dish along a grid pattern. The laser pulse rate of 50 Hz provided multiple laser pulses at any particular spot on the gel.

### Quantifying contents release

ATP concentration was measured with a commercial ATP Determination Kit (ThermoFisher, Waltham, MA), which is based on the luciferin-luciferase bioluminescence assay. Luciferase requires ATP to produce light (emission maximum ~560 nm). A standard curve was obtained with ATP standard solution, provided by the vendor. Calcium concentration was determined with a fluorescent calcium indicator, Oregon Green 488 BAPTA (Ex./Em. 494/523 nm; ThermoFisher, Waltham, MA), which exhibits an increase in fluorescence upon binding calcium ion. Fluorescence intensity was compared to a calcium standard curve.

Carboxyfluorescein (CF; ThermoFisher, Waltham, MA) is self-quenching, showing minimal fluorescence at 517 nm following excitation at 495 nm when encapsulated within liposomes at concentrations greater than 0.1 mM. Typical loading concentration of the liposomes in these experiments was 25 mM. Following liposome rupture, CF is diluted, leading to an increase in fluorescence signal at 517 nm.

Fractional release of all cargo molecules is taken relative to the solution concentration measured following complete lysis of the liposomes with Triton-X:

$$ \% \,Release=\frac{{I}_{r}-{I}_{0}}{{I}_{T}-{I}_{0}}\times 100 \% $$

in which I_r_ is the measured signal intensity following NIR irradiation, I_0_ is the background signal intensity, and I_T_ is the maximum signal intensity following liposome lysis by Triton X-100. Complete lysis was assumed when the cloudy liposome-HGN solution turned clear. Prior to irradiation, all liposomes were tested for leakage using the appropriate detection system. No significant leakage was observed for a minimum of 1 month for calcium, ATP or carboxyfluorescein.

## Supplementary information

Supporting Information.
