# Supplementary material for: Near Infrared-Triggered Liposome Cages for Rapid, Localized Small Molecule Delivery
Source: Sci Rep. 2020 Feb 3;10:1706. doi: 10.1038/s41598-020-58764-3 (PMC6997424; doi:10.1038/s41598-020-58764-3)
Supplement: Supplementary file 1 — Supporting Information. [file 41598_2020_58764_MOESM1_ESM.pdf]

Supplemental Information

Near Infrared-Triggered Liposome Cages for Rapid, Localized Small Molecule Delivery

*Jeong Eun Shin, Maria O. Ogunyankin[1] and Joseph A. Zasadzinski**

Department of Chemical Engineering and Materials Science, University of Minnesota, Minneapolis, Minnesota 55455, USA

Hollow Gold Nanoshell Synthesis Methods

The size and size distribution, and therefore the hollow gold nanoshell (HGN) local surface plasmon resonance (LSPR), are determined by the size and shape distribution of the silver nanoparticles used as sacrificial templates and the ratio of gold to silver in the subsequent galvanic replacement reaction as described in Refs. 2, 3, and the final shape cube vs sphere, of the HGN [4]. By controlling synthesis conditions, HGN of multiple different sizes can be made with tunable LSPR.

Silver Templates

This synthesis is based on that described originally in our Ref. 3. Cubic Ag templates ≥ 18 nm were prepared by heating 5 ml of diethylene glycol (DEG) in a 10 mL glass beaker to 150 °C in a silicon oil bath under magnetic stirring at 340 rpm [4,5]. After heating for 30 min, 30 mM NaSH (0.006 mL) in DEG was added. Four minutes later, 30 mM HCl (0.05 mL) in DEG was added, followed by 20 mg/ml polyvinylpyrrolidone (1.25 mL) (PVP) in DEG. After another 2 min, 282 mM CF_3COOAg (0.4 mL) in DEG.

One minute after CF_3COOAg addition the transparent solution became yellow, indicating the generation of Ag nuclei, which was accompanied by a drop in temperature to ~ 130 °C.

Monodisperse silver nanocrystals are favored by a rapid burst of nucleation of silver crystal nuclei. This burst nucleation is followed by the slow, controlled growth of the nuclei [4,5].

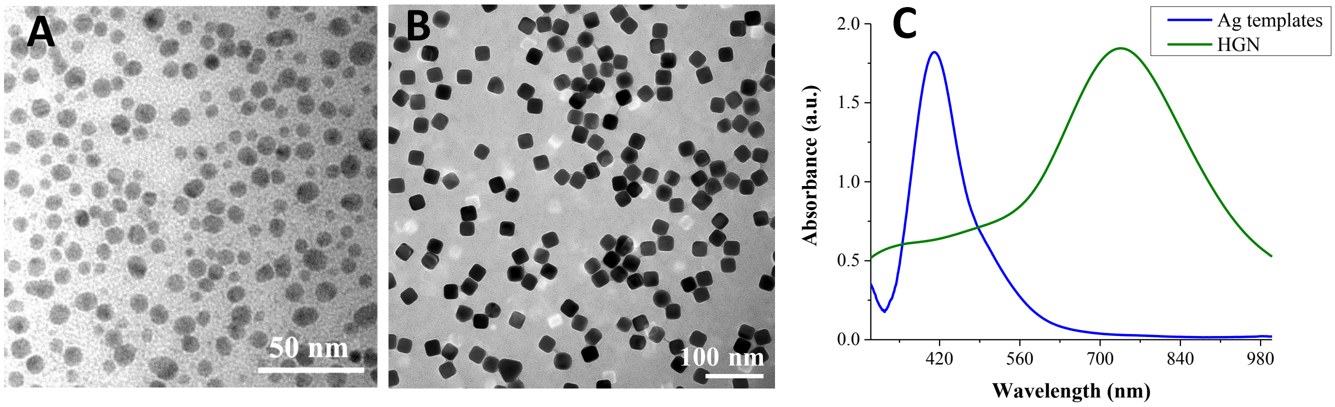

Fig. S1. Silver templates of mean size (A) 11 ± 3 nm and (B) 31 ± 3 nm. The HGN size is dictated by the template dimensions as shown in Fig. S2. The size distributions were confirmed by single particle tracking with a Nanosight instrument. (C) The silver template plasmon resonance is centered at 420 nm for all sizes of template. The SPR red-shifts on conversion to HGN depending on the relative amount of gold to silver in the galvanic replacement reaction [4].

Poly(vinylpyrrolidone) (PVP) selectively binds to the (100) silver surface, directing the growth of silver nanocrystals with cubic shapes. During growth, Ag atoms are preferentially added to the {111} facets of a single-crystal seed, leading to the formation of sharp cornered cubic nanocrystals. PVP also stabilizes the silver templates against aggregation. The reaction time determines the size of the silver templates; 30 minutes for 18 nm particles to 180 min for 32 nm particles [4,5]. (Fig. S1). The solution goes through three color changes during growth: dark reddish brown, reddish green, and bright yellowish green as the edge length of the cubic Ag seeds increased. The reaction was quenched by placing the beaker in a cold-water bath. The reaction product was washed with acetone followed by centrifugation at 4000 rpm for 30 min to remove the remaining Ag precursor and DEG, and then washed with water followed by centrifugation at 13,000 rpm for 10 min (repeated three times) to remove excess PVP. The cubic Ag nanoparticles were redispersed in DI water. The surface plasmon resonance for the silver templates was 420 – 430 nm (Fig. S1C).

For silver templates smaller than 20 nm, the oil bath was replaced by electrical resistance tape wrapped around the reaction beaker (Briskheat, Sigma Aldrich). The beaker heater keeps the

reaction temperature constant by supplying heat to the solution at a rate such that the reaction temperature of 150 °C is recovered in less than a minute following the nucleation step. The improved temperature control and uniformity promotes single burst nucleation and a monodisperse population with a mean edge length of ~9 nm for 10 minutes of reaction and ~15 nm sized Ag templates for a reaction time of 15 min (Fig. S1).

Hollow Gold Nanoshell Synthesis

HGN self-assemble by galvanic replacement of the silver templates by gold (III) chloride hydrate ($HAuCl_4$). Gold ($AuCl_4^-$/Au 0.99V, vs. SHE) has a higher redox potential than silver (Ag^+/Ag 0.8V, vs. SHE), leading to the spontaneous plating of metallic gold onto the dissolving silver template aa follows [4]:

$$3Ag_{(s)} + AuCl_{4\,(aq)}^- \rightarrow Au_{(s)} + 3Ag_{(aq)}^+ + 4Cl_{(aq)}^-$$

Ag templates in water (5 mL) were heated to 90 °C under magnetic stirring at 350 rpm and kept at 90 °C for 10 min. The stirring speed was increased to 950 rpm and the appropriate volume of 1 mM $HAuCl_4$ in water (Fig. S2C) was added dropwise into the reaction solution. The color changed from yellow to blue. The amount of $HAuCl_4$ was adjusted to fine tune the LSPR peak by following the reaction with UV-Vis spectroscopy. (Fig. S2C). On completion, the solution was cooled, silver chloride precipitated out of solution and was separated, and the supernatant containing the gold nanoshells stored at 4°C.

For a given silver template, increasing the ratio of Au to Ag red-shifts the LSPR peak (Fig. S2C), by decreasing the thickness of the shell walls [3-5] because silver is replaced by gold at a 3:1 stoichiometry. For 5 ml of the as-prepared 27 nm cubic Ag templates, adding 300 - 600 µl of 1 mM $HAuCl_4$ solution increased the LSPR absorption maximum from 600 – 900 nm (Fig. S2C). The silver templates in Fig. S1 and the HGN in Fig. S2 were prepared for transmission electron

microscopy by spreading ~ 2 μl of sample suspension as onto lacey carbon TEM grids (Electron Microscopy Sciences), allowing the solvent to evaporate and imaging with an FEI Technai Sphera G2. Silver template and HGN concentration and size distributions were measured using single particle tracking with a Nanosight NTA 2.3 particle-tracking device. The mean size given by TEM and particle tracking showed good agreement.

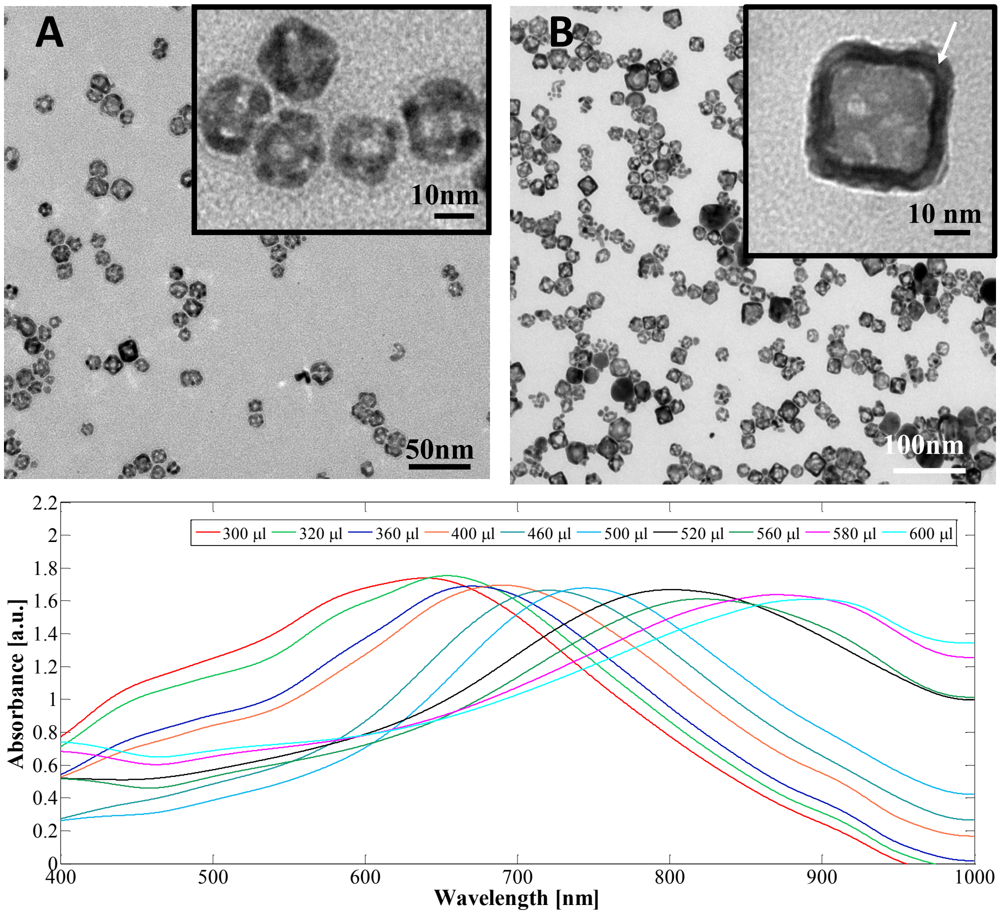

Fig. S2: A, B) Hollow gold nanoshells made from the silver templates in Fig. S1 A, B, respectively by the galvanic replacement of silver by gold. The sizes of the HGN increased 2-4 nm due to the gold shell plating on the silver template. The walls are porous so water is both inside and outside the shell [4].

C) The gold plating and hollow shape red-shifts the spectra to the NIR [4] depending on the gold to silver ratio in the synthesis For a fixed silver template size of 27 nm, adding increasing amounts of 1 mM $HAuCl_4$ red-shifts the LSPR peak from 600 - 900 nm, without significantly altering the magnitude of the peak (From Ref. 3).

Steric Stabilization of Silver and Gold Nanoparticles

To minimize aggregation during silver nanoparticle storage, sodium citrate was added to make up a 5 mM solution after washing. The citrate binds to the Ag templates and provides an electrostatic barrier to aggregation and the size distribution of the silver templates was stable for months at low ionic strength. To stabilize the HGN against aggregation in 300 mM ionic strength buffer solutions, 750 Da methoxy-PEG-thiol was added to the HGN at a ratio of 1:10 mol PEG:Au overnight at room temperature (See Fig. S3). With a PEG coating of 1:15 mol PEG:Au the nanoshells are stable overnight (12 hours), but with a denser PEG coating of 1:5 mol PEG:Au, the nanoshells are stable for weeks. Excess 750 Da PEG-thiol was removed by repeated washing and centrifugation steps. The PEGylated HGN were stable for weeks in water or saline.

Binding HGN to Liposomes

Liposomes were prepared by mixing 95:5 DPPC:DSPE-PEG-SH or 55:40:5 DPPC:cholesterol:DSPE-PEG-SH at 25 mg/ml total lipid concentration in chloroform, and the solvent was evaporated by flow of nitrogen gas in glass vials. The lipid mixtures were hydrated with the desired cargo (25 mM carboxyfluorescein (CF) in TES buffer, 10 -50 mM calcium in PBS buffer, or 10 mM ATP in PBS buffer) for 30 minutes at 60 °C. This was followed by extrusion with an Avanti Mini-Extruder through Watson 200 nm polycarbonate filters.

HGN binding was optimized by controlling the ratio of 750 Da PEG-thiol to gold during the nanoshell synthesis [1]. Insufficient PEG did not prevent flocculation in high ionic strength buffers, while too much PEG prevented liposome-HGN binding as shown in Fig. S3. Number concentrations of liposomes and HGN were determined by particle counting using a Nanosight system and HGN were added to the liposome suspensions at a 10:1 nanoshell to liposome ratio.

Our goal was to provide 1-3 HGN per liposome to ensure that all liposomes would be ruptured by a given light pulse. As shown in Fig. S3, for the 1:15 PEG:Au ratio, the HGN acted as crosslinkers and would bind and aggregate multiple liposomes. At a 1:5 PEG:Au ratio, the PEG layer prevented the HGN from binding to the liposomes. The intermediate 1:10 PEG:Au ratio provided 1-3 HGN to liposome ratio and was used for all experiments here.

Alginate Gelation

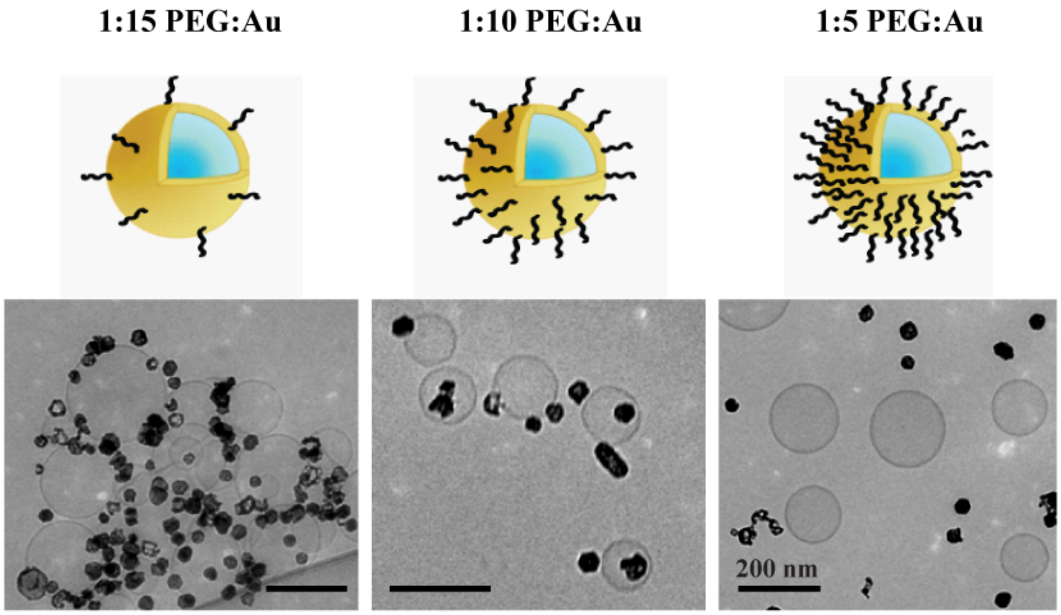

Figure S3. Impact of nanoshell PEG surface coverage on liposomal tethering. At low surface coverage, there is a high bare gold surface available for binding and cross-linking occurs between the liposomes and nanoshells. At high surface coverage, there is insufficient free surface area for the lipid-PEG-SH to bind to the nanoshell. The liposomes bind to the nanoshells at intermediate nanoshell surface coverage [1].

Alginate is a natural polysaccharide extracted from brown seaweed that consists of mannuronic and guluronic monomers. The guluronic region can crosslink with divalent cations such as Ca^{2+}; the stiffness of gel depends on the calcium concentration. To find the minimum calcium concentration for alginate gelation, alginate (Millipore-Sigma) was dissolved in PBS at 2, 4, and 8 (w/v)% and mixed with calcium chloride solutions to achieve 10 mM to 100 mM concentrations. 2%, 4%, and 8% of alginate solution showed soft gelation at 100 mM, 60 mM, and 50 mM calcium concentration, respectively. To increase the calcium levels locally, 1000 nm liposomes were made

by hydrating 50 mg/ml 95:5 DPPC:DSPE-PEG-SH with 500 mM calcium chloride and 0.4 mM of Arsenazo III calcium specific dye (Millipore-Sigma). To increase the encapsulation efficiency, 10 freeze-thaw cycles were performed, each cycle consisted of 5 minute plunges in liquid nitrogen followed by 5 minute thaws in a water bath at 65°C. The freeze-thaw cycles were followed by extrusion through micron diameter filters. 40 nm HGN with a LSPR of 800 nm were mixed with the liposomes to tether the HGN to the liposomes via the thiol linker on the DPSE-PEG-SH. Calcium containing HGN-liposomes labeled with 0.4 mM Arsenazo III dye were mixed with 8 % of alginate solution at a mixing ratio of 2:1 (Liposome:Alginate). NIR pulses at 140 mJ/cm^2 at 800 nm wavelength triggered release of calcium from the liposomes to crosslink the alginate solution. Before the irradiation, calcium was entrapped in liposomes, resulting in red color throughout the gel (See Figure 8); following irradiation, the calcium diffused away from the liposomes and complexed with the alginate, leading to white/gold colored stripes where the irradiation occurred.

An 8 wt% alginate gel, partially crosslinked with 50 mM calcium chloride, was further crosslinked by NIR irradiation of calcium containing 40 nm HGN/liposomes along four grid lines with different laser fluences (40, 30, 20, and 10mJ/cm^2), as shown in Figure 8. The gel was tilted at 30° to induce flow following irradiation and the gold colored stripes were followed (highlighted by gold lines in Fig. 8) to show that the gel stiffness could be spatially altered by different NIR fluences, which in turn led to different calcium release from the liposomes.

Cell Trapping and Growth in Irradiated Gels

A 2 % (w/v) alginate gel was mixed with PC3 cells [6] and calcium containing 40 nm HGN/liposomes with a LSPR peak of 800 nm. The alginate was partially gelled with 100 mM calcium chloride. Calcium was released from the HGN-liposomes upon irradiation with 800 nm

NIR pulses at 80 mJ/cm^2 of laser fluence. The released calcium further crosslinked the alginate gel along the irradiated grid lines. The gel was then washed with PBS and cell growth medium (RPMI1640) then incubated in cell growth medium for 48 hours. PC3 cells remained only in irradiated parts of gel after 48 hours of incubation. Cell viability was indicated by cell growth.

Shape and Size Dependence of Light Absorption

Quasistatic Approximation

Mie theory in the quasistatic limit can relate the local surface plasmon resonance (LSPR) frequency for core-shell spheres (See Figure S2C) with particle diameters much smaller than the wavelength of the incident light [2] as a function of the ratio of shell thickness, *t*, to overall size, *R* [2]. Water, with a constant, real dielectric function, ϵ_W, makes up the nanoshell core as well as the external medium. The gold-silver alloy shell has a frequency dependent dielectric function $\epsilon_G(\lambda)$. Eqns. S1- S2 give the absorption cross section, σ_{abs} [2]:

$$\sigma_{abs} = R^3 \frac{8\pi^2\sqrt{\epsilon_W}}{\lambda} Im\left(\frac{\epsilon_G\epsilon_a - \epsilon_W\epsilon_b}{\epsilon_G\epsilon_a + 2\epsilon_W\epsilon_b}\right) \qquad \text{(S1)}$$

$$\epsilon_a = \epsilon_W(3 - 2P) + 2\epsilon_G P \qquad \text{(S1a)}$$

$$\epsilon_b = \epsilon_G(3 - P) + \epsilon_W P \qquad \text{(S1b)}$$

and the shape factor, *P*:

$$P = \frac{R^3 - (R-t)^3}{R^3} \approx \frac{3t}{R} + o\left(\frac{t}{R}\right)^2 . \qquad \text{(S1c)}$$

$$\sigma_{abs} \approx (R^2 t)\left[\frac{8\pi^2\sqrt{\epsilon_W}}{\lambda}\right] Im\left(\frac{(2\epsilon_G{}^2 - \epsilon_W\epsilon_G - \epsilon_W{}^2)}{3\epsilon_G\epsilon_W}\right) \sim \xi R^2 t \qquad \text{(S2)}$$

This shows that $\sigma_{abs} \sim \xi R^2 t$, in which ξ is a frequency-dependent function of the dielectric constants of water and metal, and R^2t is proportional to the volume of the metal portion of the nanoshell. The results for the gold-silver alloy HGN are shown in Fig. S4.

Electromagnetic simulations

We used Full-field finite-difference time-domain (FDTD) electromagnetic simulations to extract the absorption cross sections [7] as described in Ref. 4. The metal alloy dielectric function was modeled as $\varepsilon(\omega) = \varepsilon_\infty - \frac{\omega_p{}^2}{(\omega^2 + i\Gamma\omega)}$, in which $\varepsilon_\infty$is the high-frequency dielectric constant, ω_p is

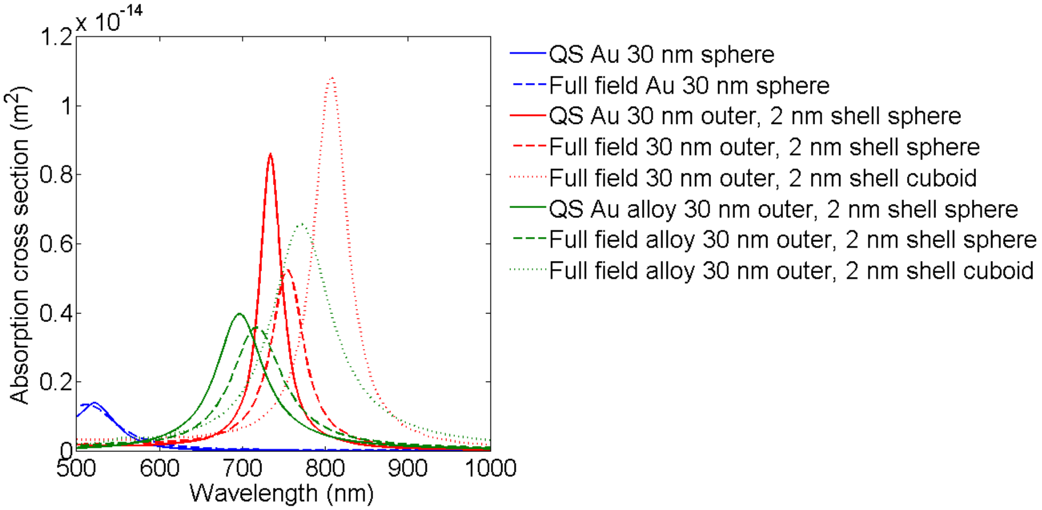

Figure S4. Comparisons of the quasistatic (QS) analytical approximation (Eqn. S1 – S3) for spherical core-shell spheres using the properties of pure Au or the 50-50 Au-Ag alloy to the full field electromagnetic simulations for spheres and cuboids. There is a small red-shift and decrease of the magnitude of the cross section for the full field calculation compared to the quasistatic approximation for spheres of the same composition. Changing from a sphere to a cube provides a much larger red-shift and a significant increase in the cross section. The alloy blue-shifts both spheres and cubes and decreases the cross section (Figure taken from Ref. 4).

the plasma frequency, and Γ is the damping parameter following the data of Peña-Rodriguez et al. [8] for gold-silver alloys. The complex permittivity as a function of silver mole fraction, x_{Ag} is $\varepsilon(\omega) = (8.6 - 4.6 * x_{Ag}) - \frac{(8.96 + 0.02x_{Ag})^2}{\omega^2 + i\omega(0.06 + 0.47x_{Ag} - 0.46x_{Ag}^2)}$ [8]. Figure S4 shows a comparison between the quasistatic analytical model predictions and those of the full FDTD simulations.

For a fixed outer diameter (or silver template diameter), increasing the ratio of gold to silver during galvanic replacement decreases the shell thickness as the stoichiometry dictates that 3 silver ions are removed per gold atom. This causes the resonance to red-shift to higher wavelengths (Fig.

S2C) [2,9]. Similarly, for a fixed shell thickness, increasing the particle diameter red-shifts the resonance to higher wavelengths (which also increases the ratio of the inner to outer radii). Cubic shapes with sharp edges are red-shifted relative to spherical shapes for the same diameter and shell thickness (Fig. S4) [10]. Therefore, the LSPR wavelength can be tuned by changing the size of the template particle or the shell thickness, or by controlling the shape of the resulting particle.

Nanobubble Generation and Detection in Flow

Nanobubbles in flow were detected by the scattering of a Helium-Neon probe laser (632.8 nm, 2 mW, Thorlabs, Inc.). The liposome suspensions were pumped through a 0.2 mm ID square, hollow glass capillary of 0.1 mm wall thickness (#8320 Vitro Tubes, VitroCom, Mountain Lakes, NJ) at controlled rates. The pump beam diameter was set to 300 μm and was tilted at 15° so the pump beam would miss the lens used to collect the light from the probe beam, which was aligned normal to the capillary. The pump beam fluence was measured by registering the image of the pump beam on the capillary and measuring the beam diameter at the sample plane with a photodetector/imaging device (Luka, Andor Technology, Northern Ireland). The pulse energy was measured using a pulse energy meter (Ophir Optronics, Ltd., Israel).

The continuous low power probe beam is scattered by the refractive index difference caused by the generation of nanobubbles around the HGN, and the light intensity measured by the photodetector is collected by an oscilloscope (Teledyne LeCroy, Wavesurfer MXs-8). A decrease in the transmitted light intensity is the characteristic signal of nanobubble generation and growth, followed by a rapid rise (100 nsec) in the transmitted intensity as the nanobubbles collapse. Nanobubbles are transient events, lasting a few hundred nanoseconds. The decrease in transmitted light intensity is due to the collective light scattering from a large number of nanobubbles being generated within the irradiated volume, rather than the scattering signal from single bubbles.

Release from liposomes was strongly correlated with nanobubble formation as described in Ref. 4.

Supplementary Information References

1 Forbes, N., Pallaoro, A., Reich, N. O. *et al.* Rapid, Reversible Release from Thermosensitive Liposomes Triggered by Near-Infra-Red Light. *Part. Part. Syst. Charact.* **31**, 1158-1167 (2014).

2 Averitt, R. D., Westcott, S. L. & Halas, N. J. Linear optical properties of gold nanoshells. *J. Opt. Soc. Am. B-Opt. Phys.* **16**, 1824-1832 (1999).

3 Sun, Y. G. & Xia, Y. N. Shape-controlled synthesis of gold and silver nanoparticles. *Science (New York, N.Y.)* **298**, 2176-2179 (2002).

4 Ogunyankin, M., Shin, J. E., Lapotko, D. O. *et al.* Optimizing the NIR Fluence Threshold for Nanobubble Generation by Controlled Synthesis of 10 - 40 nm Hollow Gold Nanoshells. *Advanced Functional Materials*, 1705272 (2018).

5 Wang, Y., Zheng, Y. Q., Huang, C. Z. *et al.* Synthesis of Ag Nanocubes 18-32 nm in Edge Length: The Effects of Polyol on Reduction Kinetics, Size Control, and Reproducibility. *J. Am. Chem. Soc.* **135**, 1941-1951 (2013).

6 Huang, X., Pallaoro, A., Braun, G. B. *et al.* Modular plasmonic nanocarriers for efficient and targeted delivery of cancer-therapeutic siRNA. *Nano Lett.* **14**, 2046-2051 (2014).

7 Taflove, A. & Hagness, S. C. *Computational Electrodynamics: The Finite Difference Time Domain Method.* 3rd edn, (Artec House, Inc., 2005).

8 Pena-Rodriguez, O., Caro, M., Rivera, A. *et al.* Optical properties of Au-Ag alloys: An ellipsometric study. *Opt. Mater. Express* **4**, 403-410 (2014).

9 Mahmoud, M. A. & El-Sayed, M. A. Gold Nanoframes: Very High Surface Plasmon Fields and Excellent Near-Infrared Sensors. *J. Am. Chem. Soc.* **132**, 12704-12710 (2010).

10 Genç, A., Patarroyo, J., Sancho-Parramon, J. *et al.* Tuning the Plasmonic Response up: Hollow Cuboid Metal Nanostructures. *ACS Photonics* **3**, 770-779 (2016).
